# Classic Bartter syndrome complicated with profound growth hormone deficiency: a case report

**DOI:** 10.1186/1752-1947-7-283

**Published:** 2013-12-30

**Authors:** Masanori Adachi, Toshihiro Tajima, Koji Muroya, Yumi Asakura

**Affiliations:** 1 Department of Endocrinology and Metabolism, Kanagawa Children’s Medical Center, Mutsukawa 2-138-4 Minami-ku, Yokohama 232-8555, Japan; 2 Department of Pediatrics, Hokkaido University School of Medicine, Sapporo 060-8635, Japan

**Keywords:** Bartter syndrome, Salt-losing tubulopathy, Hypokalemia, Gitelman syndrome, Growth failure

## Abstract

**Introduction:**

Classic Bartter syndrome is a salt-wasting tubulopathy caused by mutations in the *CLCNKB* (chloride channel Kb) gene. Although growth hormone deficiency has been suggested as a cause for persistent growth failure in patients with classic Bartter syndrome, in our opinion the diagnoses of growth hormone deficiency has been unconvincing in some reports. Moreover, Gitelman syndrome seems to have been confused with Bartter syndrome in some cases in the literature. In the present work, we describe a new case with *CLCNKB* gene mutations and review the reported cases of classic Bartter syndrome associated with growth hormone deficiency.

**Case presentation:**

Our patient was a Japanese boy diagnosed as having classic Bartter syndrome at eight months of age. The diagnosis of Bartter syndrome was confirmed by *CLCNKB* gene analysis, which revealed compound heterozygous mutations with deletion of exons 1 to 3 (derived from his mother) and ΔL130 (derived from his father). His medical therapy consisted of potassium (K), sodium chloride, spironolactone, and anti-inflammatory agents; this regime was started at eight months of age. Our patient was very short (131.1cm, -4.9 standard deviation) at 14.3 years and showed profoundly impaired growth hormone responses to pharmacological stimulants: 0.15μg/L to insulin-induced hypoglycemia and 0.39μg/L to arginine. His growth response to growth hormone therapy was excellent.

**Conclusions:**

The present case strengthens the association between classic Bartter syndrome and growth hormone deficiency. We propose that growth hormone status should be considered while treating children with classic Bartter syndrome.

## Introduction

Classic Bartter syndrome (BS), also referred to as type III Bartter syndrome, is a rare genetic disorder characterized by salt wasting from the renal tubules, mainly the thick ascending loop of Henle [1]. It is caused by mutations in the *CLCNKB* gene that encodes the type b kidney chloride channel (ClC-Kb). Patients with classic BS fail to thrive from infancy and exhibit hypokalemia, metabolic alkalosis, hyperactive renin-aldosterone system, and overproduction of prostaglandins. Although potassium supplements, anti-aldosterone agents, and/or indomethacin are the mainstay of therapy, management of growth failure and hypokalemia is still challenging [1,2].

The association of growth hormone deficiency (GHD) with classic BS has been anecdotally reported, and GHD may be one of the causes of persistent growth failure frequently observed in patients with classic BS [2-8]. However, the degrees of GHD in the reported cases have been diverse, and hence, GHD has not yet been regarded as a definite complication of BS. In addition, most of the reported cases of BS accompanying GHD were not investigated on a molecular basis [3,7,8]. Moreover, Gitelman syndrome (GS) seems to have been confused with BS in older reports in the literature [4-6]. Here, we report a case of classic BS with documented *CLCNKB* gene mutations in a boy who was found to have profound GHD. We also present a literature review on the association between classic BS and GHD.

## Case presentation

Our patient was a Japanese boy born at 41 weeks of gestation via spontaneous cephalic delivery, with a birth weight of 3,680g. His family history was remarkable in that his elder sister, who was five years older than him, had been diagnosed as having classic BS when she was five months old: her final height was 147.0cm (−2.1 standard deviation [SD]) and at a recent assessment her insulin-like growth factor 1 (IGF-1) level was 286ng/mL (normal range for her age, 168 to 459ng/mL).

**Figure 1:**
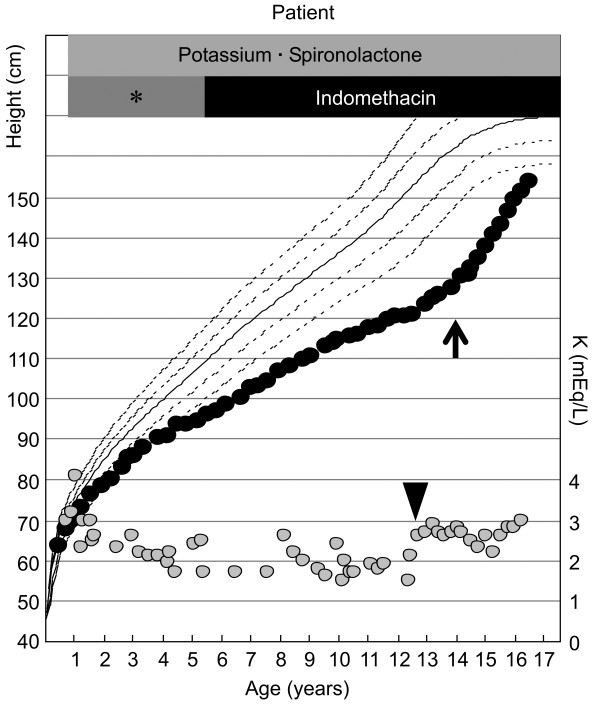
**Growth charts of our patient superimposed with variations in his serum potassium levels.** Black circles indicate heights; gray circles indicate potassium levels. The arrowhead indicates the age at which our patient could ingest potassium tablets, allowing higher potassium levels than before. Arrow indicates the initiation of growth hormone therapy. *Non-indomethacin anti-inflammatory agents such as tolmetin sodium and mefenamic acid.

At eight months of age, our patient was diagnosed as having classic BS based on the following findings: failure to thrive, metabolic alkalosis (pH 7.423; HCO_3_^-^, 33.6mmol/L; base excess, +8.2), hypokalemia (2.9mEq/L), and hyperactive renin-aldosterone system (plasma renin activity (PRA), 270ng/mL/h; normal value for his age, 2.58 ± 1.41ng/mL/h); aldosterone level, 850pg/mL (2,358pmol/L; normal value for his age, 173.7 ± 96.3pg/mL). The diagnosis of BS was confirmed by *CLCNKB* gene analysis, which revealed compound heterozygous mutations with deletion of exons 1 to 3 (derived from his mother) and ΔL130 (derived from his father), the latter of which has been reported previously by the authors TT and MA. Medical therapy consisting of potassium (K), sodium chloride, spironolactone, and anti-inflammatory agents was initiated at eight months of age and is still ongoing. However, as depicted in Figure 1, his serum K level remained considerably low because he was unable to consume large amounts of drugs, especially potassium preparations. Our patient also displayed mild intellectual impairment: he could only speak meaningful words by the age of three, and required specialized primary education.

When he was 11 years old, an investigation for macrohematuria led to the detection of renal stones with nephrocalcinosis. This complication resolved following the amelioration of hypokalemia, which was achieved by our patient’s increased efforts to ingest potassium tablets.

**Figure 2:**
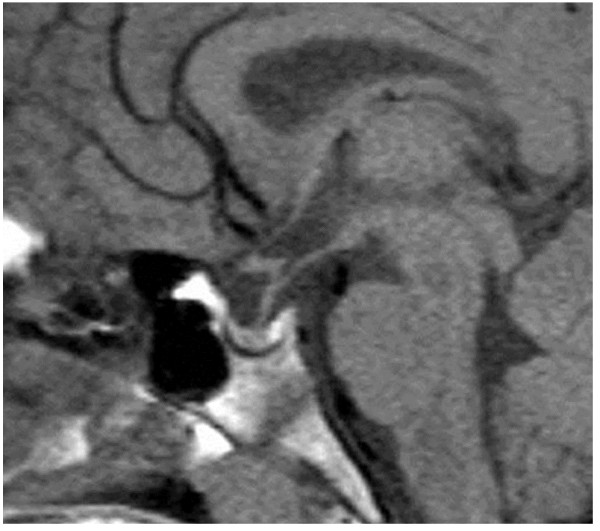
Magnetic resonance imaging scan of the pituitary gland of our patient.

At 14.3 years of age, his severe short stature (131.1cm, -4.9SD) prompted us to evaluate his growth hormone (GH) status, and he was found to have profound GHD. His serum levels of IGF-1 and IGF binding protein 3 were 80ng/mL (normal range for his age, 178 to 686ng/mL) and 1.92μg/mL (normal range for his age, 2.69 to 4.16μg/mL), respectively. Pharmacologically stimulated GH levels were 0.15 and 0.39μg/L after insulin-induced hypoglycemia and arginine administration, respectively (Table 1). His bone age was 11.4 years (Tanner-Whitehouse 2-radius, ulna and short bones (TW2-RUS) method for Japanese individuals). Magnetic resonance imaging study results revealed no abnormalities in the hypothalamic-pituitary region (Figure 2).

**Table 1:** Results of pharmacological growth hormone stimulation tests in our patient at 14 years of age

	**0 minutes**	**30 minutes**	**60 minutes**	**90 minutes**	**120 minutes**
Insulin-induced hypoglycemia:					
Blood glucose (mg/dL)	94	54	93	99	92
Growth hormone (μg/L)	0.11	0.07	0.15	0.13	0.08
Arginine:					
Growth hormone (μg/L)	0.11	0.26	0.39	0.28	0.17

GH therapy was initiated at 14.5 years of age at a dose of 21 to 27μg/kg/day, which restored his growth remarkably (Figure 1). Although his pubertal stage progressed from Tanner stage 1 to stage 2 over the next two years, his bone maturation (Δbone age/Δchronological age) was 1.02. No significant change was observed in his serum potassium level during GH therapy.

## Discussion

To the best of our knowledge, the association of BS with GHD was first reported in 1977 [3]. Thereafter, a number of similar reports have been published [2-8]. However, we believe that some of the older cases reported in the literature do not comply with the current definition and concept of BS and thus should be recognized as GS [4-6]. GS is another salt-losing tubulopathy caused by mutations in the *SLC12A3* gene that encodes the thiazide-sensitive sodium-chloride cotransporter (NCCT) [1]. Because classic BS and GS shared the laboratory finding of hypokalemic alkalosis, these conditions were not strictly discriminated until the era of molecular diagnosis. Molecular diagnosis is a prerequisite for the detailed study of classic BS.

Tables 2 and 3 summarize cases of GHD reported to date classified as BS [2,3,7-10] and GS [4-6,11-16], respectively. Our patient’s case is remarkable in that the diagnosis of classic BS was established molecularly. In addition, our patient’s GH responses to pharmacological stimulants were most profoundly impaired among the hitherto reported cases. Although one may argue that hypokalemia may blunt the GH response and lead to false negative results, the excellent response to GH therapy made us suspicious for the presence of GHD. By adding our patient to the existing list of cases of GHD concomitant with BS, we believe that GHD should be regarded as a complication in classic BS.

**Table 2:** Classical Bartter syndrome with growth hormone deficiency: cases from the literature

**Reference**	**Age, years**	**Sex**	**Mutation**	**GH peak (μg/L) to stimulants**	**IGF-1 (ng/mL)**
[9]	5	M	IVS2-1G > C/W610X	9.3 (GLC), 8.0 (CLN), 8.2 (l-DOPA), 38.0 (ARG)	Not determined
[10]	8	F	Not determined	2.9 (INS), 2.0 (CLN), 6.9 (GRF)	122.1
[7]	10	M	Not determined	3.20 (INS), 3.20 (l-DOPA)	25
[8]	10	F	Not determined	0.70 (l-DOPA), 1.96 (CLN)	41.5
	11	M	Not determined	4.70 (l-DOPA), 1.79 (CLN)	39.7
	11	M	Not determined	0.50 (l-DOPA), 4.49 (CLN)	38.3
[2]	11	M	ΔExon1-6/ΔExon1-6	7.6 (ARG)	Low
	14	M	ΔExon1-19/ΔExon1-19	2.4 (ARG), 8.4 (GRF)	Low
[3]	22	F	Not determined	Absence (INS), 8.0 (ARG)	Not determined
Present case	14	M	ΔL130/ΔExon1-3	0.15 (INS), 0.39 (ARG)	80

*ARG* arginine, *CLN* clonidine, *l-DOPA*l-3,4-dihydroxyphenylalanine, *GH* growth hormone, *GLC* glucagon, *GRF*, growth hormone releasing factor, *IGF-1* insulin-like growth factor 1, *INS* insulin.

**Table 3:** Gitelman syndrome (including definite or probable cases) and GHD: cases from the literature

**Reference**	**Age, years**	**Sex**	**Mutation**	**GH peak (μg/L) to stimulants**	**IGF-1**
[12]	3	M	2614fr/unknown (*SLC12A3*)	<8 (INS), <8 (ARG), <8 (CLN)	Not determined
	9	F	G186D/unknown (*SLC12A3*)	6 (CLN)	89ng/mL
[5]	3	M	Not determined	3.3 (l-DOPA), 7.3 (CLN)	0.26U/mL
	9	F	Not determined	9.2 (l-DOPA), 4.8 (CLN)	0.67U/mL
	19	F	Not determined	6.0 (CLN)	Not determined
[6]	7	M	Not determined	9.8 (INS + ARG)	Not determined
[11]	9	M	Not determined	2.1 (INS), 3.2 (CLN), 1.8 (l-DOPA)	55ng/mL
[13]	10	F	Not determined	7.5 (l-DOPA), 6.9 (CLN)	Normal
[14]	11	M	Not determined	10.8 (GRF), 7.0 (CLN)	0.43U/mL
[15]	11	M	Not determined	5 (INS), 1 (CLN), 13 (GRF)	292ng/mL
[4]	11	M	Not determined	11 (CLN), 3.1 (GLC)	0.74U/mL
[16]	13	M	Not determined	5.4 (INS), 5.4 (ARG), 12 (GLC-PPL)	0.19U/mL

Cases were categorized as Gitelman syndrome according to the authors’ own judgment, even if they were described as Bartter syndrome in the original reports.

*ARG* arginine, *CLN* clonidine, *l-DOPA*l-3,4-dihydroxyphenylalanine, *GH* growth hormone, *GLC* glucagon, *GRF* growth hormone releasing factor, *IGF-1* insulin-like growth factor 1, *INS* insulin, *PPL* propranolol.

Flyvbjerg *et al.* suggested that hypokalemia is a causative factor of GHD [17]. These authors stated that mice fed a low potassium diet showed growth retardation with low IGF-1 levels and attenuated GH response to GH-releasing factor (GRF). From this observation, hypokalemia seems to be one of the possible factors responsible for GHD in classic BS. This hypothesis is strengthened by the findings that GHD has also been reported in other diseases predisposing to hypokalemia, such as GS (Table 3) and the Bartter-like Dent disease [18]. In addition, this hypothesis can help to differentiate GHD (our patient in the present report) from non-GHD (his sister). Because large amounts of potassium could be administered via the gastric tube or tablets, a higher serum potassium level could be maintained in the sister, which may have prevented the development of GHD. Furthermore, the lack of association between GHD and antenatal BS, which is caused by mutations in either the *SLC12A1* (type I BS) or *KCNJ1* (type II BS) gene, can be explained by the observation that the correction of hypokalemia is generally easier in antenatal BS than in classic BS.

However, factors other than hypokalemia may be necessary for developing GHD. Patients with familial aldosteronism, rare genetic forms of primary aldosteronism, present with hypokalemia and some of them are refractory to medical therapy, yielding to long standing hypokalemia [19]. Regardless, GHD has not been reported to date in patients with familial aldosteronism. Thus, an aim of our future studies would be to determine the precise mechanism by which GHD develops in patients with classic BS.

## Conclusions

In summary, we report our experience of profound GHD in a boy with mutations in the *CLCNKB* gene, and propose that GH status should be monitored while treating salt-losing tubulopathies including classic BS and GS.

## Consent

Written informed consent was obtained from the patient’s next-of-kin for publication of this case report and any accompanying images. A copy of the written consent is available for review by the Editor-in-Chief of this journal.

This study was approved by the Institutional Review Board of Kanagawa Children’s Medical Center and followed the World Medical Association Declaration of Helsinki regarding ethical conduct of research involving human subjects.

## Abbreviations

BS: Bartter syndrome; GH: Growth hormone; GHD: GH deficiency; GRF: GH-releasing factor; GS: Gitelman syndrome; IGF-1: Insulin-like growth factor 1.

## Competing interests

The authors declare that they have no competing interests.

## Authors’ contributions

MA treated our patient from the beginning, performed the *CLCNKB* gene analysis and evaluated the GH status of our patient. MA also wrote the manuscript. TT and KM planned and performed the *CLCNKB* gene analysis. YA and KM critically reviewed and revised the manuscript. All authors read and approved the final manuscript.
